# Editorial: Photobiomodulation and phototherapy in skin diseases

**DOI:** 10.3389/fmed.2023.1357286

**Published:** 2024-01-09

**Authors:** Michael R. Hamblin, Xiang Wen, Bingrong Zhou

**Affiliations:** ^1^Laser Research Centre, Faculty of Health Sciences, University of Johannesburg, Doornfontein, South Africa; ^2^West China Hospital, Sichuan University, Chengdu, China; ^3^Department of Dermatology, Nanjing Medical University, Nanjing, China

**Keywords:** dermatology, lasers - therapeutic use, acne (acne vulgaris), cosmetic, phototherapy

Skin is the organ which is most extensively exposed to a broad range of wavelengths of light. Photobiomodulation and phototherapy are becoming promising therapeutic approaches for treating a wide range of cutaneous diseases and disorders, including psoriasis, acne, atopic dermatitis, hair regrowth, wound healing, skin rejuvenation, and pigmentation disorders. Light-based approaches have the advantages of being non-pharmaceutical, easily applied, cost-effective and non-invasive. Various light sources have been investigated, including lasers, intense pulsed light, light-emitting diode arrays, and UV sources. The wavelengths employed range from UV, blue, green, yellow, red to near-infrared. Although some of the light sources are pulsed, others are continuous wave mode. Power levels can vary from highly focused lasers that cause microscopic thermal damage to low-level light that triggers cellular signaling pathways. Some light sources are designed to target specific biological chromophores in the tissue, including blood vessels, melanin, cellular mitochondria, and light-activated ion channels. Different biological structures, including stem cells, immune cells, hair follicles, collagen, and bacteria colonizing the skin can be affected. The strong growth in the practice of Cosmetic Dermatology as world populations have grown in affluence and the usage of social media, has encouraged the use of phototherapy approaches, because there is an understandable reluctance of many patients to take drugs to improve their cosmetic appearance.

This Research Topic was designed to foster insights into advances in the mechanisms and application of photobiomodulation and phototherapy for skin diseases in order to provoke further translational research in this area. We eventually ended up with two original research papers, two brief research reports, one clinical trial and one case report.

The first research report was by Marchegiani et al. who investigated the use of fluorescent light energy (FLE) to treat canine superficial bacterial folliculitis. Superficial bacterial folliculitis (SBF) is a bacterial infection that is confined within the hair follicles, without invasion of the dermis (1). *Staphylococcus pseudintermedius* is a normal commensal species in canine skin and is the main cause of SBF (2). Topical and systemic antibiotics are the most often employed treatments for canine SBF, but antibiotic resistance is a growing problem. FLE consisted of applying a layer of yellow gel containing fluorescent dyes to the affected skin and then irradiating with blue light (440 nm−460 nm, 55–129 mW/cm^2^) for 2 min. Six dogs received FLE once, six dogs twice, while eight dogs received oral antibiotics until complete healing was observed. FLE was able to significantly reduce the time needed for clinical resolution compared to antibiotics alone, improving the owners' compliance and welfare of the dogs.

The second research report was by Hu et al. who carried out a retrospective evaluation of hemoporfin-mediated photodynamic therapy (HM-PDT) for port-wine stains. Hemoporfin is hematoporphyrin monomethyl ether and has been shown to be effective in destroying the abnormal vasculature after IV administration and activation by green light (3). Because PDT can be very painful, general anesthesia is sometimes employed in patients who would otherwise be unable to tolerate the procedure. This paper asked the question whether the use of general anesthesia affected the outcome of the PDT on the port wine stain. They looked at 207 patients treated with HM-PDT of whom 137 received a general anesthetic, while 69 did not. The treatment efficacy was significantly higher in the general anesthetic group than in the non-anesthetic group (76.81% vs. 56.52%, *p* < 0.05). Purpura lasted longer in the anesthetic group, but the other side effects were similar between the two groups.

Solar lentigenes are disfiguring brown spots on the face, which are often treated with a picosecond pulsed 755-nm alexandrite laser especially in Asian skin types (4). In a brief research report, Liu et al. asked whether video education could improve the process of obtaining informed consent for this procedure. In a retrospective study they compared 56 patients who received additional video education, while 50 patients underwent traditional informed consent. More correct answers were given by older and less educated patients who received video education, while the video group also reported improved patient satisfaction compared to the traditional group.

The second brief research report by Yu et al. described a prospective randomized half-body study comparing 308 nm LED light vs. 308 nm excimer laser for treating localized psoriasis. The 308 nm excimer laser has been used to treat psoriasis since 2000 (5), but the device is expensive and bulky. Ten patients with symmetrical skin lesions of mild-to-moderate psoriasis completed a prospective, randomized, split sided clinical trial. The target lesions were randomly treated with either LED light or excimer laser twice a week for 12 weeks. The responses as evaluated by the local psoriasis severity index scores and dermoscopic features were similar on both sides of the body. Because 308 nm LEDS are more portable and cost-effective compared to the excimer laser, they should be considered in the future for psoriasis treatment.

The clinical trial was conducted by Dai et al. who investigated the laser treatment of atrophic acne scars in Asian patients. Atrophic acne scars are a disfiguring and distressing complication of acne which may affect up to 95% of patients (6). Dai et al. carried out a 20-week prospective, randomized, split-face, controlled pilot study comparing 1064-nm Nd:YAG picosecond laser using a fractional micro-lens array (P-MLA) vs. an ablative fractional 2940-nm Er:YAG laser (AF-Er) in 31 patients. The Echelle d'Evaluation Clinique des Cicatrices d'acne (ECCA) grading scale, Investigator Global Assessment (IGA) scores, patient satisfaction, and VISIA analysis were employed to evaluate the improvement in the scars. Both lasers produced equivalent improvements in ECCA and IGA scores, but patient satisfaction was higher for the AF-Er-treated side. VISIA analysis revealed the pore and skin texture was similar for both devices. No serious side effects were reported, but the P-MLA side had less pain and shorter duration of crust shedding and edema.

The final case report by Ping et al. described the treatment of a patient with acne vulgaris using a combination of 5-aminolaevulinic acid mediated photodynamic therapy and the monoclonal antibody adalimumab. Adalimumab (also known as Humira) binds to and inactivates the pro-inflammatory cytokine, tumor necrosis factor alpha (TNF-a) (7). It has used to treat many chronic inflammatory and autoimmune diseases, such as rheumatoid arthritis, psoriasis, Crohn's disease, and ulcerative colitis (8), but has not often been used as a monotherapy for acne (9). ALA-PDT is often used for treating recalcitrant acne due to its ability to reduce sebum secretion while at the same time killing the *Cutibacterium acnes* bacteria, which colonize the sebaceous glands (10). The patient who suffered from severe acne and had previously failed other therapies, was treated with ALA PDT once a week for 3 weeks and three injection of Humira once every 2 weeks. The active acne lesions then resolved leaving some residual scars.

We hope that these papers in the present Research Topic will encourage other clinicians to further explore the wide range of phototherapy options for skin diseases, including sophisticated lasers and more economical LEDs, as well as simple lamps.

## Author contributions

MH: Writing—original draft, Writing—review & editing. XW: Writing—original draft, Writing—review & editing. BZ: Writing—original draft, Writing—review & editing.
